# Supplementary figures and images for: Soil moisture dynamics under two rainfall frequency treatments drive early spring CO2 gas exchange of lichen-dominated biocrusts in central Spain
Source: PeerJ. 2018 Nov 16;6:e5904. doi: 10.7717/peerj.5904 (PMC6241396; doi:10.7717/peerj.5904)

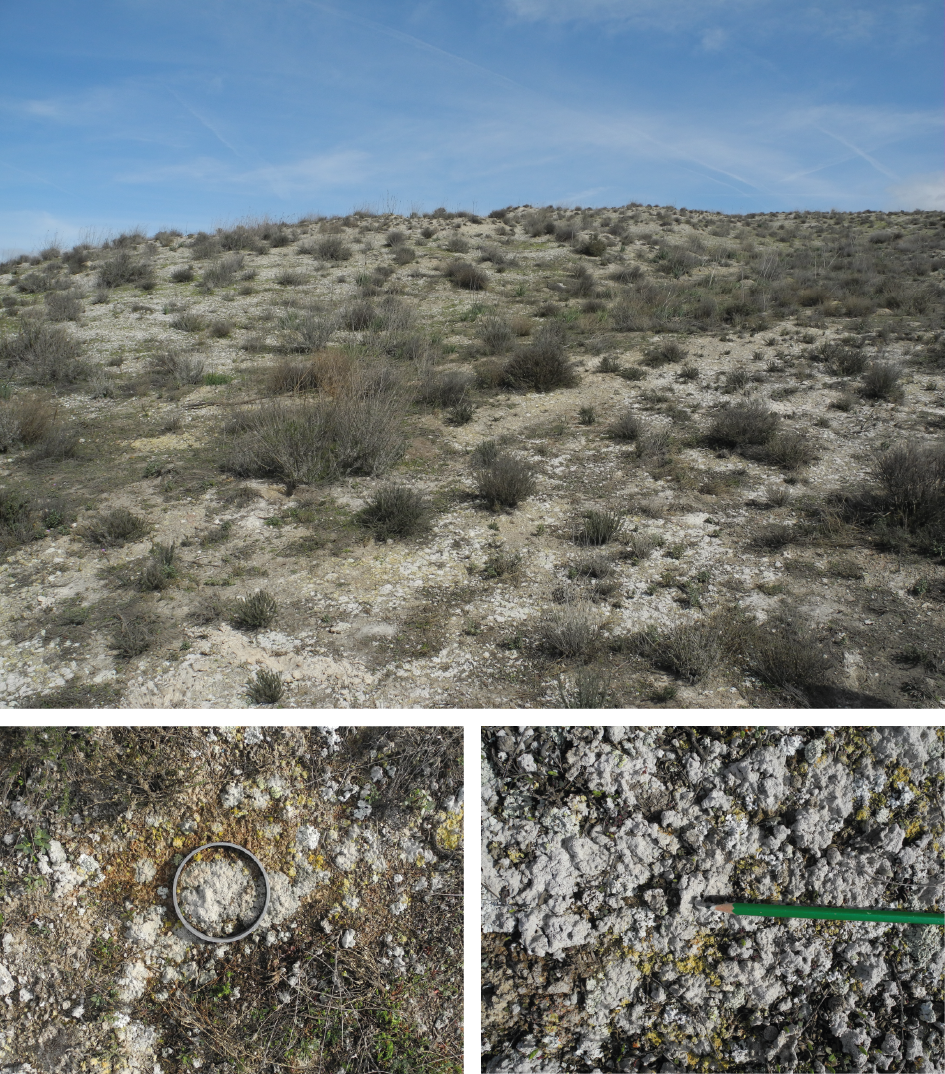

Supplement: Supplemental Information 1 — The lichen-dominated biocrusts cover a substantial part of the interplant space and are characterized by a high cover of Diploschistes diacapsis (bottom right) (Photo credit: Selina Baldauf). [file peerj-06-5904-s001.png]

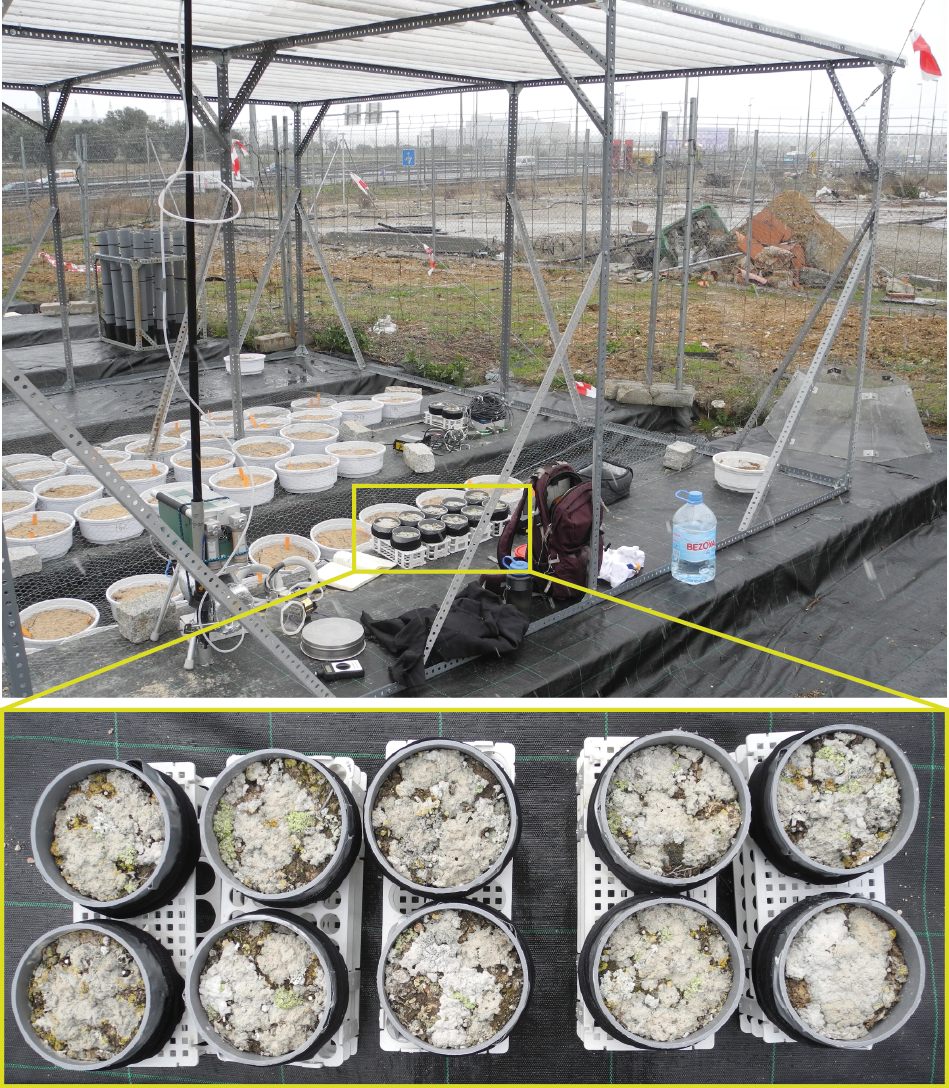

Supplement: Supplemental Information 2 [file peerj-06-5904-s002.png]

— Relative humidity — Dewpoint temperature • Lichen surface temperature

**2017-03-21**

**2017-03-22**

**2017-03-23**

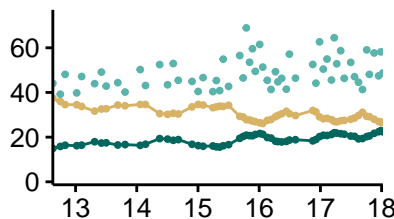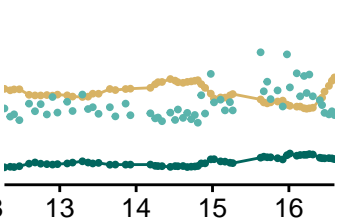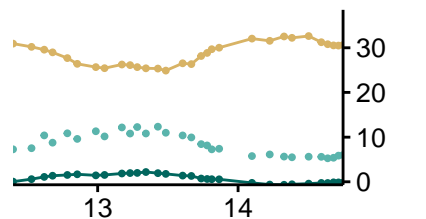

**2017-03-24**

**2017-03-25**

**2017-03-26**

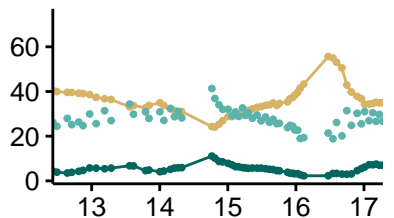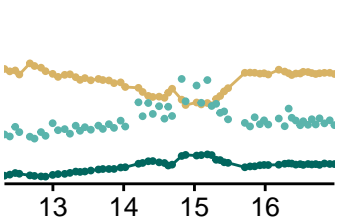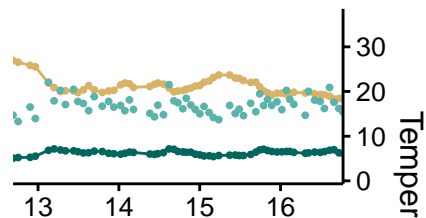

**2017-03-27**

**2017-03-28**

**2017-03-29**

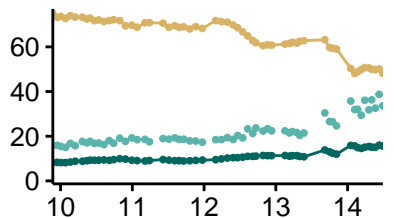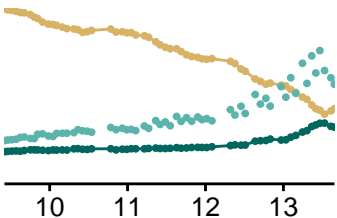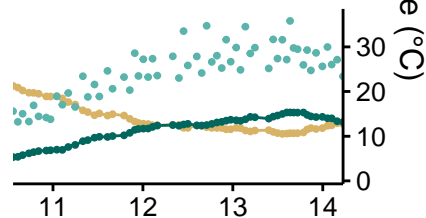

**2017-03-30**

**2017-03-31**

**2017-04-01**

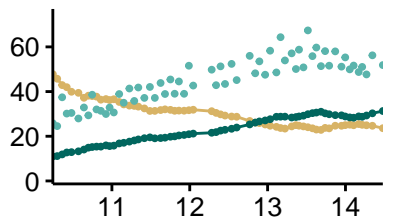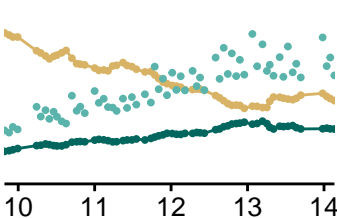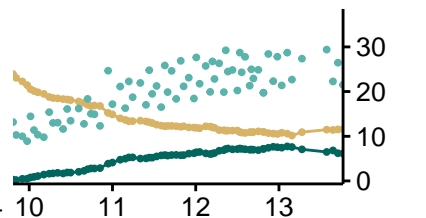

Solar hour

Supplement: Supplemental Information 3 — During the measurements the lichen surface temperature was always higher than the dewpoint temperature, i.e. no dew occurred during the measuring. [file peerj-06-5904-s003.pdf]

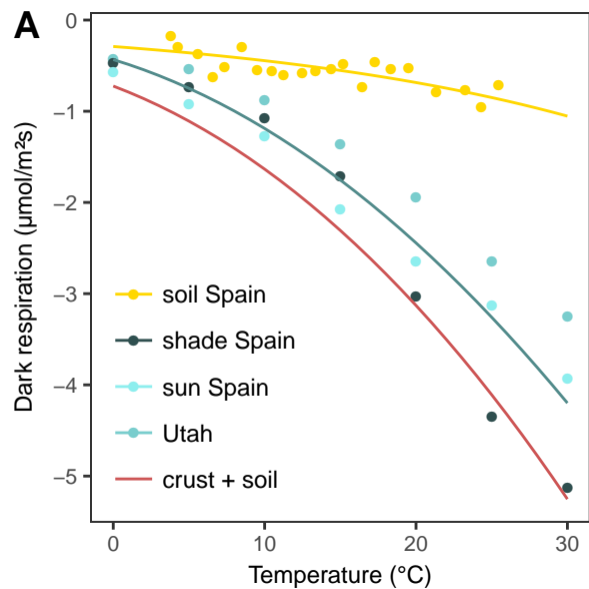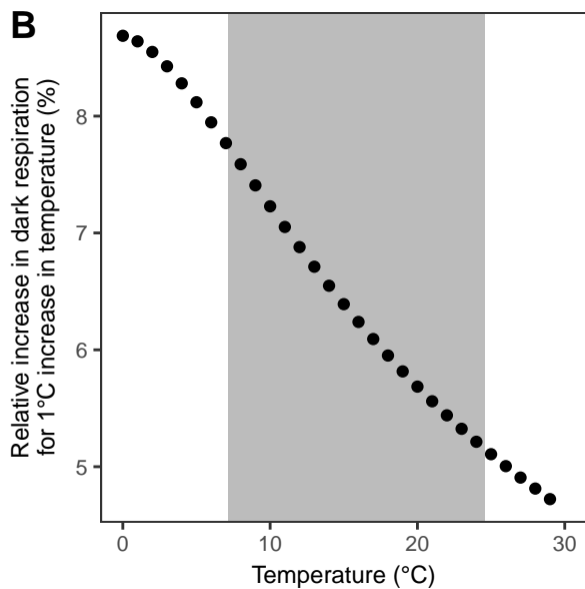

Supplement: Supplemental Information 5 — (A) Temperature dependency of respiration. Crust data is from a population in Utah (Lange et al., 1997) and a sun and shade population in Spain (Pintado et al., 2005), both measured at optimal water content. Bare soil respiration data is from a study conducted in Aranjuez (central Spain) with soil similar to the soil used in this study at soil moistures above 25%. The blue and yellow lines are fits for crust and soil respiration, the red line is the additive respiration of soil and crust. (B) Relative increase in crust and soil dark respiration for a 1°C increase in temperature as calculated from the red line in 2A. The grey box indicates the soil temperature relevant to our study in which relative increases in dark respiration of ca. 5-8% are expected. [file peerj-06-5904-s005.pdf]
