# Supplementary material for: Soil moisture dynamics under two rainfall frequency treatments drive early spring CO2 gas exchange of lichen-dominated biocrusts in central Spain
Source: PeerJ. 2018 Nov 16;6:e5904. doi: 10.7717/peerj.5904 (PMC6241396; doi:10.7717/peerj.5904)
Supplement: Supplemental Information 4 — The blue area indicates that the high frequency flux was higher and the orange area indicates that the low frequency flux was higher. The red dots represent the mean. The asterisks show significant differences between the two treatments, calculated with a paired Wilcoxon rank sum test (p < 0.05*, p < 0.001***, ns = not significant, n = 60 for 0 and 1 days since the last watering and n=50 for 2 days since the last watering). [file peerj-06-5904-s004.pdf]

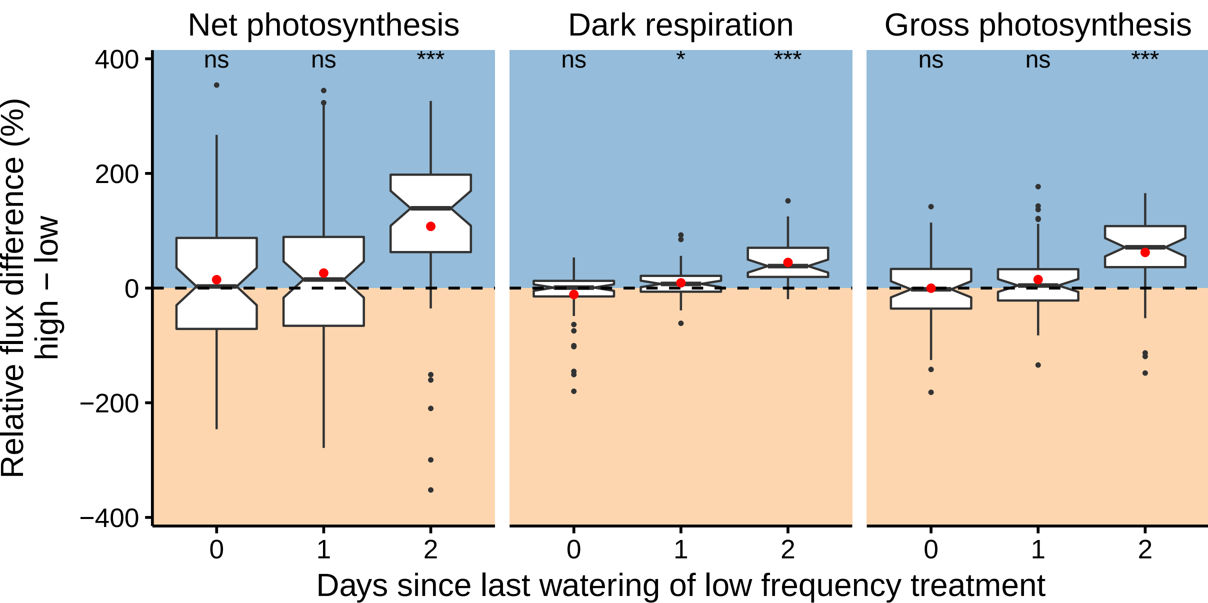

Net photosynthesis
Dark respiration
Gross photosynthesis
ns
ns
***
ns
*
***
ns
ns
***
400
200
0
−200
−400
0
1
2
0
1
2
0
1
2
Relative flux difference (%)
high − low
Days since last watering of low frequency treatment
