# Supplementary material for: Soil moisture dynamics under two rainfall frequency treatments drive early spring CO2 gas exchange of lichen-dominated biocrusts in central Spain
Source: PeerJ. 2018 Nov 16;6:e5904. doi: 10.7717/peerj.5904 (PMC6241396; doi:10.7717/peerj.5904)
Supplement: Supplemental Information 6 [file peerj-06-5904-s006.pdf]

daily measurement no	watering treatment	days since last watering	mean time since last watering (h)
1			1.5
2	5 mm/day	0	3.1
3			4.5
1			1.3
2	15 mm/3 days	0	3.0
3			4.5
1			24.8
2	15 mm/3 days	1	26.3
3			27.8
1			49.2
2	15 mm/3 days	2	50.9
3			52.0
