# Supplementary material for: Soil moisture dynamics under two rainfall frequency treatments drive early spring CO2 gas exchange of lichen-dominated biocrusts in central Spain
Source: PeerJ. 2018 Nov 16;6:e5904. doi: 10.7717/peerj.5904 (PMC6241396; doi:10.7717/peerj.5904)
Supplement: Supplemental Information 7 — Note that on March 21st, no moisture measurement was available, therefore, the cumulative moisture is lower than in the other periods. [file peerj-06-5904-s007.pdf]

Period	cumulative moisture 5 mm/day (%)	cumulative moisture 15 mm/3 days (%)
March 21st - March 23rd	33.7	33.1
March 24th - March 26th	73.6	70.1
March 27th - March 29th	75.9	68.6
March 30th - Aprilst	58.0	51.4
