# Supplementary material for: Soil moisture dynamics under two rainfall frequency treatments drive early spring CO2 gas exchange of lichen-dominated biocrusts in central Spain
Source: PeerJ. 2018 Nov 16;6:e5904. doi: 10.7717/peerj.5904 (PMC6241396; doi:10.7717/peerj.5904)
Supplement: Supplemental Information 8 — Effects of the watering treatments and photosynthetically active radiation (PAR) or soil temperature (Tsoil) on net photosynthesis, dark respiration and gross photosynthesis. Significant values are highlighted in bold. [file peerj-06-5904-s008.pdf]

Response	Source	Chisq	Df	Pr(>Chisq)
**Net photosynthesis** ~	watering treatment	0.09	1	0.767
	PAR	82.02	1	<**0.001**
	watering treatment:PAR	8.44	1	**0.004**
**Dark respiration** ~	watering treatment	4.05	1	**0.044**
	T_{soil}	322.41	1	<**0.001**
	watering treatment:T_{soil}	9.93	1	**0.002**
**Gross photosynthesis** ~	watering treatment	4.87	1	**0.027**
	PAR	358.10	1	<**0.001**
